# Clinical pharmacokinetics of quinine and its relationship with treatment outcomes in children, pregnant women, and elderly patients, with uncomplicated and complicated malaria: a systematic review

**DOI:** 10.1186/s12936-022-04065-1

**Published:** 2022-02-10

**Authors:** Teerachat Saeheng, Kesara Na-Bangchang

**Affiliations:** 1 Center of Excellence in Pharmacology and Molecular Biology of Malaria and Cholangiocarcinoma, Chulabhorn International College, Thammasat University, Rangsit Campus, 99 Moo 18 Phaholyothin Road, Klong Luang District, Pathumthani, 12121 Thailand; 2 Drug Discovery and Development Center, Office of Advanced Science and Technology, Thammasat University, Pathumthani, Thailand

**Keywords:** Pharmacokinetics, Quinine, Pregnancy, Children, Elderly, Systematic-review

## Abstract

**Background:**

Standard dosage regimens of quinine formulated for adult patients with uncomplicated and complicated malaria have been applied for clinical uses in children, pregnant women, and elderly. Since these populations have anatomical and physiological differences from adults, dosage regimens formulated for adults may not be appropriate. The study aimed to (i) review existing information on the pharmacokinetics of quinine in children, pregnant women, and elderly populations, (ii) identify factors that influence quinine pharmacokinetics, and (iii) analyse the relationship between the pharmacokinetics and treatment outcomes (therapeutic and safety) of various dosage regimens of quinine.

**Methods:**

Web of Sciences, Cochrane Library, Scopus, and PubMed were the databases applied in this systematic search for relevant research articles published up to October 2020 using the predefined search terms. The retrieved articles were initially screened by titles and abstracts to exclude any irrelevant articles and were further evaluated based on full-texts, applying the predefined eligibility criteria. Excel spreadsheet (Microsoft, WA, USA) was used for data collection and management. Qualitative data are presented as numbers and percentages, and where appropriate, mean + SD or median (range) or range values.

**Results:**

Twenty-eight articles fulfilled the eligibility criteria, 19 in children, 7 in pregnant women, and 2 in elderly (14 and 7 articles in complicated and uncomplicated malaria, respectively). Severity of infection, routes of administration, and nutritional status were shown to be the key factors impacting quinine pharmacokinetics in these vulnerable groups.

**Conclusions:**

The recommended dosages for both uncomplicated and complicated malaria are, in general, adequate for elderly and children with uncomplicated malaria. Dose adjustment may be required in pregnant women with both uncomplicated and complicated malaria, and in children with complicated malaria. Pharmacokinetics studies relevant to clinical efficacy in these vulnerable groups of patients with large sample size and reassessment of MIC (minimum inhibitory concentration) should be considered.

**Supplementary Information:**

The online version contains supplementary material available at 10.1186/s12936-022-04065-1.

## Background

Parenteral artesunate, followed by artemisinin-based combination therapy (ACT), is the recommended treatment for severe malaria, when available. Quinine is usually used if artesunate is not available, and is always used in combination with an additional agent, such as doxycycline [1]. Quinine is also used, in combination with clindamycin, for uncomplicated *Plasmodium falciparum* infections in pregnant women during the first-trimester, as well as for chloroquine-resistant *Plasmodium vivax* infections [1]. A systematic review in 2012 revealed that artesunate was superior to quinine in reducing mortality rate and adverse drug reactions in severe malaria [2]. A recent systematic review (2020) has suggested a high risk of treatment failure in uncomplicated malaria patients (mostly second- and third-trimester pregnancies) after quinine monotherapy, while the efficacy of quinine-clindamycin combination was comparable with ACT [3].

The current dosage regimens of quinine for children, elderly, and pregnant women, based on adults’ regimens, may be suboptimal due to altered physiology and pharmacokinetics [4]. None of the current systematic reviews address the impact of the differences in pharmacokinetics on quinine efficacy and safety in these vulnerable populations [5, 6]. Pharmacokinetic investigation of quinine may be required for further dosage optimization in these populations. The objective of this study is to provide a systematic review of the existing information on the dose regimens and pharmacokinetics of quinine in children, pregnant women, and elderly populations, and to determine whether modification of dosage regimens in these populations is required.

## Methods

### Data sources

The Web of Science, Cochrane Library, Scopus, and PubMed were used by two independent researchers as search databases up to October 2020. The search terms included ‘Quinine’ AND ‘Pharmacokinetics’ AND ‘Pregnancy’ OR ‘children’ OR ‘Pediatrics’ OR ‘Neonates’ OR ‘Newborns’ OR ‘Infants’ OR ‘Geriatrics’ OR ‘elderly’ (> 65 years).

### Inclusion and exclusion criteria

The inclusion criteria were: the article published in English, and the article involved clinical pharmacokinetic research on quinine in either children, elderly, or pregnant women.

### Data extraction and quality assessment

TS and PK independently retrieved and reviewed articles, and any argument or inconsistency was resolved by the third person (KN). The information extracted were: area under the plasma drug concentration–time curve (AUC), maximum plasma concentration (C_max_), time to reach maximum plasma concentration (t_max_), trough plasma concentration (C_trough_), elimination or terminal half-life (t_1/2_ or t_1/2β_), apparent volume of distribution (V_d_, V_d_/F, V_ss_, or V_ss_/F), total clearance (CL or CL/F), efficacy, and toxicity parameters. The AUC, C_max_, and C_trough_ were normalized with body weight. As there are different values of half-life, volume of distribution, and clearance parameters reported in various studies, the full descriptions of parameter terms are used in the articles. Qualitative data are presented as number and percentage (%). Quantitative data are summarized as mean, mean ± SD, median, median (range), or range values. The volume of distribution and clearance for the intramuscular (im), intrarectal (ir), and oral (po) doses are reported by adjusting to an absolute bioavailability (F) of 0.95, 0.60, and 0.76, respectively.

Quality of the article selection process was assessed using the checklist for the assessment of the methodological quality for both randomized and non-randomized studies of health care intervention [7].

## Results and discussion

**Fig. 1:**
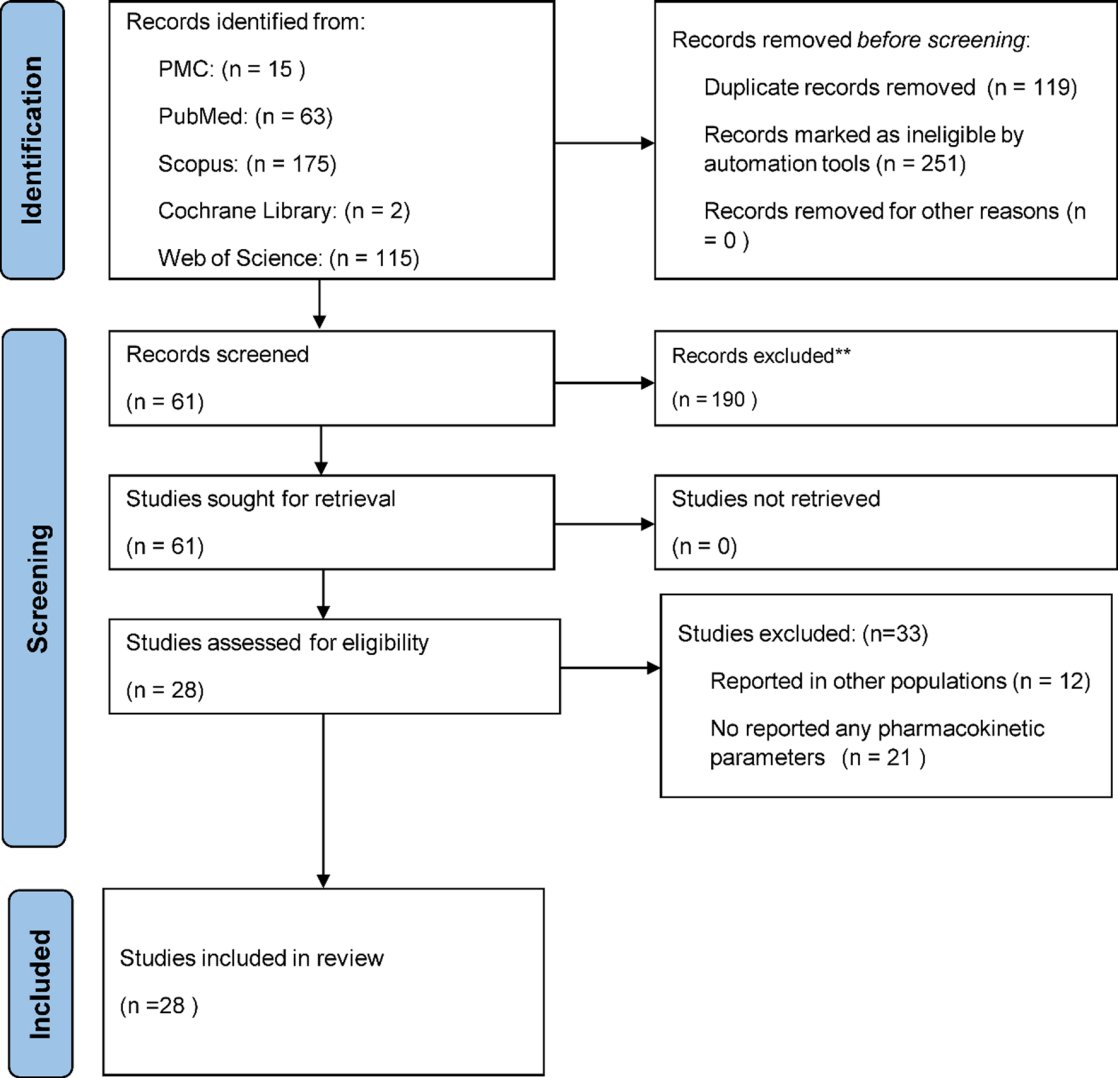
A schematic workflow of systematic review of quinine used in vulnerable subjects

Three hundred and seventy articles were retrieved from the databases, and 28 articles were selected for further analysis based on the predefined criteria (Fig. 1). The average scores for quality assessment were acceptable. Nineteen, 7, and 2 articles involved studies in children [8–26], pregnant women [27–33], and elderly [34, 35], respectively. The studies were conducted during 1982–2013 in 16 countries; 23, 2, and 3 studies in Africa, Asia, and Australia/Oceania, respectively. Fourteen, 11, and 3 studies involved uncomplicated falciparum malaria [8–16, 27–31], complicated falciparum malaria [17–25, 32, 33], and non-malaria [9, 11, 14], respectively.

Three pharmacokinetic analysis approaches were applied i.e., non-compartmental analysis (NCA, n = 13) [10, 16–19, 22, 26–28, 30–32, 34], compartmental-analysis (CA, n = 11) [8, 9, 11–13, 15, 20, 21, 23, 33, 35], and population-based pharmacokinetic analysis (pop-PK, n = 4) [14, 24, 25, 29]. NCA is the standard method which provides pharmacokinetic parameters directly from the observed data giving a gross approximation as the result, and is not the most accurate method for parameter estimation. This analysis approach however, is not suitable for the characterization of pharmacokinetic variability in the populations. CA offers the advantage of allowing for the use of data modeling and simulation for dosage optimization. The pop-PK approach is most applicable to analysis of drug pharmacokinetics in various populations considering both intra- and inter-individual variability. Additional file 1: Table S1–3 summarizes quinine pharmacokinetics (focusing on C_max_ and systemic exposure) reported in children, pregnant women, and elderly with uncomplicated and complicated malaria.

### Dose-dependent pharmacokinetics

Three out of 28 articles (10.71%) reported the pharmacokinetic parameters of both quinine and the active metabolite 3-hydroxyquinine (2 and 1 in pregnancy and children, respectively) [10, 27, 28]. For quinine, the AUC, C_max_, and C_trough_ were reported in 20 (71.4%) [8–13, 17–29, 31, 34], 26 (82.1%) [8–13, 15–23, 25–29, 31–35], and 16 (57.1%) [8–13, 15–17, 19, 21, 23, 26, 29–31] articles, respectively. The corresponding numbers for 3-hydroxyquinine were 2 (66.7%), 3 (100%), and 1 (33.3%) article(s), respectively. Since the anti-malarial potency of 3-hydroxyquinine is tenfold lower than quinine [36], measurement of plasma concentrations and analysis of pharmacokinetic parameters may be unnecessary.

#### Children

AUC_0-8 h_ (mg/l/h) data were reported in 7 articles following different routes and dosage regimens; 5 in uncomplicated and 2 in complicated malaria (1 in malnourished children). The ranges of body-weight adjusted AUC_0-8 h_ in uncomplicated and complicated malaria were 0.97–2.47 [8–13] and 3.10–3.74 [17–23, 25] mg/l/h/kg, respectively.

Malaria severity, nutritional status, administration route, and body weight are the key factors influencing quinine exposure and treatment outcomes. With iv dose administration, AUC_0-8 h_ following 4.74 mg base/kg in uncomplicated and complicated malaria were 1.2–2.47 [8, 9, 13] and 3.1–3.74 23 mg/l/h/kg, respectively. The impact of malaria severity on quinine exposure is explained by the significantly lower rate of clearance in cases of complicated malaria (0.06 ± 0.04 l/h/kg) [18–20, 23–25] compared with uncomplicated (0.09 l/h/kg) malaria [14] and healthy subjects (0.24 ± 0.14) [23]. Down-regulation of the drug-metabolizing enzyme cytochrome P450 (CYP) has been reported in *Plasmodium chabaudi-*infected mice [37]. Kwashiorkor [26], but not malnutrition [10, 23], had a significant effect on quinine exposure. With equivalent dosage, the AUC_0-8 h_ following the im and ir routes were contradictory, being either higher [8, 9] or comparable [17–20] with the iv route. The ir route provided the lowest AUC_0-8 h_ [23], and a threefold increase in quinine dose (base) [11] may be required to obtain an AUC_0-8 h_ comparable to the iv route [8]. The AUC_0-8 h_ following both the im and iv, but not ir route, increased linearly with dosage.

Following 7–16 mg base/kg, the C_max_ in complicated malaria (0.4–1.22 µg/ml/kg) [8–13, 15, 16], was relatively higher than in uncomplicated malaria (0.34–0.95 µg/ml/kg) [17–23, 25]. Following 4.74 mg base/kg, the iv route provided the highest C_max_ (iv: 0.36 ± 0.07; im: 0.25 ± 0.11; ir: 0.17 ± 0.11 µg/l/kg) [8, 11]. The mean C_max_ values in nourished and malnourished children were 0.5 and 0.67 µg/ml/kg, respectively [10]. C_max_ showed a linear relationship with dose size following ir and im administration. Simulated C_max_ in children weighing 5 kg was 15.9% lower than those weighing 20 kg. However, this difference was unlikely to significantly affect quinine efficacy since plasma quinine concentrations in 95% of the patients remained within the therapeutic range [25].

#### Pregnant women

AUC data was reported in pregnancy with uncomplicated malaria in only 4 articles [27–29, 31]. A comparison of quinine pharmacokinetics between pregnant and non-pregnant women was reported in 2 articles [27, 28] wherein the AUC_0-inf_ for both groups were comparable (8.24 ± 2.43 vs. 9.90 ± 2.50 µmol/l/h) [27]. Physiological changes during pregnancy and estimated gestational age (EGA) did not appear to significantly affect quinine exposure [29]. It is noted, however, that the number of participants in each group (7 non-pregnant and 16 pregnant women) is small and may not provide adequate statistical power to allow for an accurate conclusion on the effect of pregnancy on quinine pharmacokinetics.

C_max_ was reported in all articles, 5 and 2 articles in uncomplicated and complicated malaria, respectively (0.07–0.22 vs. 0.19 µg/ml/kg) [27–29, 31]. Malaria infection and routes of administration, but not pregnancy status, had significant influence on quinine C_max_ [27]. The C_max_ following iv and po routes were 0.20 ± 0.028 [27, 28] and 0.09 ± 0.026 [29, 31] µg/ml/kg, respectively.

#### Elderly

A study in healthy elderly following a single oral dose of 600 mg quinine salt (497 mg base) [34] showed a significantly higher AUC in elderly compared with adults (1.58 ± 0.39 vs. 1.20 ± 0.32 µg/ml/kg), while the C_max_ values were comparable [34].

### Dose-independent pharmacokinetics

#### Children

Quinine clearance was reported in 8 studies (po, iv, and im), 2 in uncomplicated [10, 14], and 6 in complicated [19, 20, 23–25] malaria. Malaria infection and body weight influenced quinine clearance in children [25]. The clearance in healthy subjects and uncomplicated malaria was significantly higher than complicated malaria (0.24 ± 0.13 vs. 0.15 (0.08–0.25) vs. 0.06 (0.029–0.08) l/h/kg) [10, 14, 19, 20, 23–25]. The effect of nutritional status on quinine clearance is contradictory and needs further investigation. The clearance in malnourished children with complicated malaria was slightly higher than in nourished children (0.08 ± 0.04 vs. 0.07 ± 0.04 l/h/kg) [23]. In uncomplicated malaria, the clearance in malnourished children was significantly higher than in nourished children (0.25 ± 0.20 vs. 0.13 ± 0.08 l/h/kg) [10]. Plasma protein levels (albumin, alpha1-acid glycoprotein, or AAG) and protein-binding in both groups were similar [10]. The higher clearance observed in malnourished children with uncomplicated malaria may be due to changes in drug-metabolizing enzyme activity rather than plasma protein binding. Route of administration had no effect on total quinine clearance (0.03–0.07 [19, 20, 23] and 0.035–0.07 [18, 24, 25] l/h/kg for iv and im routes, respectively). There was no linear relationship between quinine clearance and malaria parasite density [17].

The volume of distribution of quinine was reported in 8 articles, 6 in complicated [17, 19, 20, 23–25] and 2 in uncomplicated malaria [10, 14] (0.53–1.5 vs. 1.4–2.0 l/kg). Body weight was a significant covariate for the volume of distribution [25]. The effect of malaria disease severity on volume of distribution was contradictory. The volume of distribution in cerebral malaria reported by Pussard et al. [23] was threefold lower than that reported by Frank et al. [17] (0.53 ± 0.1 vs. 1.44 ± 0.01 l/kg, respectively), but was similar to adult patients with cerebral malaria (0.74 ± 0.30 l/kg) [38]. The decrease in the volume of distribution with increasing malaria severity could be due to an increase in AAG and dehydration in patients, and/or a decrease in tissue blood flow in complicated malaria. Nutritional status had less contribution (Vd: 0.58 ± 0.22 vs. 0.53 ± 0.1 l/kg for malnourished vs. nourished children with malaria) [23].

Three out of 19 studies reported models with covariates [14, 24, 25]. None provided the stratification of age along with pharmacokinetic analysis. Age (range: 6 months to 6.7 years) was not a significant covariate that influenced clearance and/or volume of distribution. Since the activities and expressions of UDP-glucuronosyltransferase 1A1 (UGT1A1) and cytochrome P450 3A4 (CYP3A4), both major quinine metabolizing enzymes, are likely to be steady in individuals aged over 6 months [39], and 24 months [40], respectively, age is unlikely to be a significant covariate. In contrast, body-weight was a significant covariate that influenced both clearance and volume of distribution. It was clear that quinine dosage should be administered based on body-weight rather than age.

The half-life values of quinine reported in healthy, uncomplicated, and complicated malaria were 6.1 ± 1.5 [23], 7.2 ± 3.3 [9, 11, 14], and 11.6 ± 4.6 [17, 19–21, 24, 25] h, respectively. The half-life in complicated malaria was 16.8 ± 3.1 h [18, 20, 24]. It is noted that quinine half-life is prolonged in direct proportion to severity of infection. Additionally, its half-life is also influenced by nutritional status (malnutrition and kwashiorkor) [23, 26].

Quinine t_max_ in uncomplicated and complicated malaria ranged from 1.7 to 4.0 h [8–11, 13] and 1.1 to 4 h [18–21], respectively. The im route provided the shortest t_max_ (iv: 2.7–4.7 [8, 9, 11–13, 17, 19, 20, 23] vs. im: 1.0–2.7 [8–10, 13, 17–19, 21] vs. ir: 1.7–4.8 [8, 9, 11–13, 21] vs. po: 1.5–3 [14, 15] h). The delayed t_max_ seen in malnourished children compared with nourished children (6.3 ± 1.8 vs. 2.3 ± 2.9 h) [23], similar to that found with chloroquine, could be due to intestinal malabsorption [41].

#### Pregnant women

Quinine clearance during pregnancy was reported in 2 articles in uncomplicated [29–31] and 1 article in complicated [33] malaria (0.11 ± 0.04 vs. 0.07 l/h/kg). Based on pop-pK analysis, the presence of an effect from EGA status on quinine clearance was unlikely, despite the positive correlation between the EGA associated-pregnancy period and quinine clearance [29]. Since CYP3A4 activity consistently increases throughout gestation, it may not have a sizeable enough impact to be a considered a covariate. Furthermore, the initial body temperature was a significant covariate that influenced clearance due to an increase in hepatic enzyme activity. In patients presenting high body temperature upon admission, plasma concentration of quinine should be monitored. Quinine volume of distribution was reported in 3 articles, 2 in uncomplicated [29, 31] and 1 in complicated malaria [33] (2.4 ± 1.3 vs. 0.96 ± 0.27 l/kg). The reported volume of distribution was inconsistent. The volume of distribution values in uncomplicated malaria, based on pop-PK analysis, during the second and third-trimesters were 3.05 (2.66–4.26) and 3.05 (2.75–3.86) l/kg [29], respectively. The volume of distribution estimated based on NCA in pregnant women with uncomplicated malaria was 1.1 (0.62–1.97) l/kg [31], which was comparable to adult patients with uncomplicated malaria (1.26 ± 0.26 l/kg) [42]. The demographic data (e.g., age, body-weight, gestation age, and trimester of pregnancy) of the patients from the two studies were not different [29, 31]. However, it is clear that plasma volume in pregnancy during the first, second, and third trimesters increases up to 106%, 129%, and 149% of that reported in non-pregnant women [43]. In addition, an increase in fat composition, extracellular fluid, and total body water during pregnancy may result in an increased volume of distribution as quinine is moderately lipophilic. The reported volume of distribution based on NCA analysis is likely to be inaccurate.

Quinine half-life was reported in 3 articles, 2 in uncomplicated [27–29, 31] and 1 in complicated [33] (9.8–16.1 vs.11.2 + 4.3 h) malaria. Malaria severity was unlikely to affect quinine half-life during pregnancy [28]. The half-life seen in pregnant women with uncomplicated malaria, complicated malaria, and during convalescence were 12.7 ± 2.1 [28, 29, 31], 11.2 ± 4.3 [33], and 11.9 ± 1.3 h [28], respectively. These results are in agreement with that reported in non-pregnant adults with cerebral malaria, uncomplicated malaria, and during convalescence (18.2 ± 9.7 vs. 11 ± 4.1 vs. 16.0 ± 7 h) [42]. It is noted that the estimation of quinine half-life in pregnant women may be inaccurate due to the limited number of studies reporting this data.

Physiological changes during both pregnancy and malaria infection are unlikely to have affected quinine t_max_. The t_max_ was reported only in uncomplicated malaria in 3 articles, i.e., pregnant [27–29, 31] and non-pregnant [27, 28] women with uncomplicated malaria [2 (1.8–3) vs. 2.3 (2–2.5) h], which was similar to that of the convalescent phase [2 (2–4) h] [28].

#### Elderly

Quinine clearance was reported in elderly subjects [34] and elderly diabetic patients [35]. The elderly [age: 70 (65–78) y] had a reduced clearance compared with young adults [24 (20–35) y] (0.045 ± 0.007 vs. 0.06 ± 0.02 l/h/kg, respectively), but with comparable renal clearance (0.01 ± 0.003 vs. 0.012 ± 0.005 l/h/kg, respectively) [34]. Since renal clearance of quinine makes up about 20% of total clearance, the reduction of glomerular filtration rate (GFR) with age is unlikely to affect quinine clearance [44]. The diabetic status had no effect on quinine clearance (0.049 ± 0.42 vs. 0.05 ± 0.02 l/h/kg for diabetic vs. non-diabetic subjects) [35].

There was no effect of diabetic status on quinine volume of distribution (1.29 ± 0.42 vs. 1.29 ± 0.45 l/kg for non-diabetic vs. diabetic subjects) [35]. The volume of distribution in the elderly was lower than adults [44], due mainly to a decrease in total body water and cardiac output.

The prolongation of quinine half-life in the elderly compared with young adults (19.2 ± 1.1 vs. 10.5 ± 1.6 h) was consistent with the decrease of quinine clearance [34].

Quinine t_max_ was unaffected by age (2.3 ± 1.2 vs. 2.5 ± 0.7 h in elderly vs. young adults) [34], which was at least in part explained by the unchanged gastric emptying rate in the elderly. It is noted that the reports of the gastric emptying rate change in elderly, reported as either delayed [45] or unchanged [46], are discordant. Demographic data (i.e., body-weight, height, body-mass index (BMI), sex, hypertensive status, and glycosylated haemoglobin level) were not evaluated as model covariates.

### Ethnicity

Ethnicity can influence quinine clearance due to associated polymorphisms in drug-metabolizing enzymes. Twenty-three studies were carried out in Africans; the other 5 studies were carried out in Cambodians, New Zealanders, and Australian. There were no clinical studies that reported across different ethnic groups on the same malaria severity and vulnerable populations. The conclusion of the influence of ethnicity on quinine exposure is, therefore, limited. However, the distribution of genetic polymorphisms, including those in genes coding for drug-metabolizing enzymes, varies with ethnicity, suggesting the possibility that ethnicity could influence quinine clearance. The allele frequencies of CYP3A4*3, CYP3A4*13, CYP3A4*18, and CYP3A4*19 were 0.021 in European-Americans, 0.011 in Han-Chinese, 0.01 in Japanese, and 0 in Africans [47]. The proportions of patients with impaired quinine clearance in individuals with either CYP3A4*3, CYP3A4*13, CYP3A4*18, or CYP3A4*19 and those with the wild-type genotype CYP3A4*1A were 22.6%, 5.8%, 17.6%, and 43%, respectively [48]. In addition to the CYP3A4/5 polymorphisms in African subjects, four additional polymorphisms including CYP3A4*1B, CYP3A5*3, CYP3A5*6, and CYP3A5*7 were reported. The allele frequencies for CYP3A4*1B, CYP3A5*3, CYP3A5*6, and CYP3A5*7 were 0.66–0.86, 0.04–0.81, 0.05–0.25, and 0–0.21, respectively [67]. All polymorphisms except CYP3A41*B are classified as poor metabolizer (PM) due to a reduction or non-detection of enzyme expression [67]. The decrease in enzyme activity and clearance may result in toxicity. However, results from a recent physiologically-based pharmacokinetic (PBPK) modelling study suggested that the effects of these genetic polymorphisms on quinine exposure and clearance were not clinically significant and dosage adjustment may not be required [49].

### Therapeutic relevance

The clinical efficacy of different dosage regimens and routes of administration in uncomplicated malaria was excellent with a 100% cure rate [8, 9, 11–16]. It is noted that most articles reported 7-day [8, 9, 11–14] or 14-day [15, 16], but not 42-day, cure rates as recommended by the World Health Organization (WHO) for the evaluation of clinical efficacy of drugs for *P. falciparum* treatment. Three out of the 9 articles reported 100% cure rates in complicated malaria [20, 21, 23]. Susceptibility of *P. falciparum* to quinine has been decreasing over time [50–55]. Quinine plasma concentrations should be maintained above the minimum inhibitory concentration (MIC) throughout the 7-day treatment period to completely eliminate the malaria parasite. In 1983, the reported MIC in children with uncomplicated malaria in Africa was 0.37 µmol/l (0.12 µg/ml) [50], but by 1988 it had increased to 0.8 (0.16–5.12) µmol/l (0.26 (0.05–1.66) µg/ml) [51]. During the same period, the MIC reported in *P. falciparum* isolates in Thailand (Thai-Cambodian border) was 3.04 µmol/l (0.98 µg/ml) [52]. Quinine IC_50_ (concentration that inhibits parasite growth by 50%) values in adults with uncomplicated malaria in Western and Eastern Cambodia gradually increased from 2001 to 2007 (Additional file 1: Table S4) [53]. During the same period, the IC_50_ reported at the Thai-Myanmar and Thai-Cambodia borders had markedly increased (Additional file 1: Table S4) [54]. Besides the IC_50_ and MIC, mean cut-off concentration of schizont maturation (MCOC equivalent to MIC) of quinine reported in 2009 in adult patients with uncomplicated malaria in Northwestern Thailand was 36.5 µmol/l (11.84 µg/ml) [55].

#### Children

Sixteen out of 19 articles reported clinical efficacy of quinine, 8 each in uncomplicated and complicated malaria. Quinine doses in a 3-day or 5-day regimen for uncomplicated malaria during the period of 1993–2005 varied from 4.74 to 11.85 mg base/kg [8, 9, 11–14]. Since 2008, the recommended dose has been replaced with 8 mg base/kg (q8h) with a 7-day treatment course [15, 16]. During 1993–2002, the FCT (fever clearance time) following a dosage of 4.74–11.85 mg base/kg was within 36 h [8, 9, 11, 12], but has been prolonged up to 72 h since 2004 [13]. With the same dosage regimen, the FCT following the iv was faster than ir route (48 vs. 72 h) [13]. The reported influence of dose on FCT was inconsistent between reports, being either comparable for all doses (within 36 h) [11], or shortening as dose increased [13]. An increase in dose could reduce FCT due to faster parasite killing (dose response curve). The PCT (parasite clearance time) reported in uncomplicated malaria following 4.74–11.85 mg base/kg was 48–96 h [8, 9, 11–16]. The PCT was shorter following iv administration compared with the ir route (48 vs.72 h) [12]. Two articles however, reported similar PCT data with different routes (iv, im, and ir) and dosage regimens [8, 13]. Notably, other contributing factors, e.g., initial parasitemia and body-weight, were not taken into consideration during the analysis, which could be unaddressed confounding factors that resulted in incorrect data interpretation [8, 13].

Quinine doses used in complicated malaria treatment varied from 4.74 to 16.35 mg base/kg (with or without a loading dose). Four out of 8 articles reported both FCT [19, 21–23] and PCT [19, 21, 22, 24]. The FCT and PCT values in complicated malaria were 25.1–48.6 and 27.4–49.5 h, respectively [19, 21–23]. The FCT following im administration [19, 22] was shorter than the ir [21] route (27.4 ± 3.6 vs. 48.6 ± 2.7 h). This was in agreement with the relatively short t_max_ following im administration compared with ir routes. Time to regain consciousness was 36–39 h [19]. A recent systematic review and meta-analysis however, concluded no significant difference in PCT, FCT, mortality rate, duration of hospitalization, and time-to-drinking between IR and IM administration [56], but not for the effect of dosage.

Fifteen out of the 19 articles reported C_trough_ at different time points, 8 articles in uncomplicated [8–13, 15, 16] and 6 articles in complicated [17–19, 21, 23, 25] malaria, and 1 article in kwashiorkor [26]. Therapeutic ranges of total quinine concentration for children with complicated and uncomplicated malaria in Africa before and during 1994 ranged from 0.2 to 2.0 µg/ml [57], and 0.21–0.35 µg/ml unbound quinine [11], respectively. Despite the continuous decline in sensitivity of *P. falciparum* to quinine over time, the current analysis suggests that total C_trough_ in most studies in both complicated and uncomplicated malaria were maintained above the MIC for 7 days with a clinical efficacy of 100%. Total quinine C_trough_ during 1983–2010 in uncomplicated malaria ranged from 1.42 to 10.43 µg/ml (0.1–0.73 µg/ml free quinine) [8, 9, 11–13, 15, 16]. Results of pharmacokinetic/pharmacodynamic modeling reported in 2003 suggested a total C_trough_ of at least 3.4 µg/ml (0.34 µg/ml free quinine, f_u_ = 0.109 [58]) for curative treatment of uncomplicated falciparum malaria in Thai adults [59]. The consensus meeting in 2007 concluded that the required C_trough_ for uncomplicated malaria was 10–12 µg/ml 1 (1.09–1.31 µg/ml free quinine) [60], which is equivalent to 9.5–11.50 µg/ml total C_trough_ in children (f_u_ = 0.114, 1.08–1.3 µg/ml free concentrations) [14]. The therapeutic C_trough_ of quinine in children with uncomplicated malaria is therefore, 9.5–11.50 µg/ml. The dosage regimens that provided the required total C_trough_ in children with uncomplicated malaria were a 7-day course of multiple doses of 8 [16] or 10–12 [15] mg base/kg q8h for iv administration and 12 h for im administration. In addition, the suggested ir regimen was multiple doses of 11.84 mg base/kg q8h. Such routes and dose frequency of quinine administration also contribute to quinine exposure and therapeutic outcome. Dosage adjustment in children with uncomplicated malaria may not be required [Table 1], which is applicable for quinine use in malaria endemic areas where quinine sensitivity is still sufficient. In addition to improved patient compliance, im administration appears most preferable due to its lower frequency of drug administration [Table 1].

**Table 1:** A summary of promising quinine dose regimens used in clinical studies during 1982 to 2013 in vulnerable subjects

Population	Dose regimen	Required dose adjustment	
		Uncomplicated malaria	Complicated malaria
Children	8–12 mg base/kg iv q8h for 7d 8–12 mg base/kg im q12h for 7d	N	Y*
Pregnant women	8.3 mg base/kg PO q8h for 7d Loading dose of 16.7 mg base/kg iv over 4 h, followed by 8.3 mg base/kg q8h	Y -	- Y
Elderly	8.3 mg base/kg PO q8h for 7d Loading dose of 16.7 mg base/kg iv over 4 h, followed by 8.3 mg/kg q8h until patients can swallow then complete oral dose regimen	N** N**	N** N**

*Iv* intravenous infusion, *im* intramuscular injection, *PO* *per* oral

*No standard regimen of quinine administration reported for complicated malaria; **the results were estimated from a single dose of 600 mg of quinine administration in elderly

For complicated malaria, the therapeutic range of quinine for adults reported in 1983 was 5–10 µg/ml (0.35–0.7 µg/ml free quinine) [61] or 10–15 µg/ml (0.7–1.05 µg/ml unbound quinine) [62]. Quinine C_trough_ during 1982–2010 in complicated malaria ranged from 2.7 to 12 µg/ml (0.15–0.66 µg/ml free quinine) [17–19, 21, 23, 25]. The predicted C_trough_ based on data analysis from uncomplicated malaria during 1948–1995 was 8–15 µg/ml (0.72–1.05 µg/ml unbound quinine) for curative treatment of complicated malaria [25]. The suggested therapeutic C_trough_ in children with complicated malaria is therefore 14.4–19.09 µg/ml (f_u_ = 0.055 [20], 0.72–1.05 µg/ml free quinine). None of the dosage regimens reported in the current analysis provided sufficient C_trough_ for treatment of complicated malaria in children [Table 1]. It is noted that the recommended standard dose regimen for complicated malaria was not used in the included articles. Pharmacokinetic/pharmacodynamic studies of quinine in children with complicated malaria following standard dose regimens are required to evaluate their effectiveness.

#### Pregnant women

Four out of 7 articles reported clinical efficacy of quinine, 3 in uncomplicated [28, 30, 31] and 1 in complicated [32] malaria during pregnancy. PCT was reported in 1 study (24–57 h) [30]. None of the included studies reported FCT. The cure rates in uncomplicated and complicated malaria were 99.2% [28, 31], and 91.67% [32], respectively. Two quinine regimens were used for uncomplicated malaria, i.e., multiple oral doses of 8.3 mg base/kg q8h for 7 days [29–31], and a single oral dose followed by artemether (ACT) [27]. In complicated malaria, a standard dose regimen (a loading dose of 16.7 mg base IV over 4 h, followed by 8.3 mg base/kg IV over 4 h q8h) was applied [32, 33]. Three articles reported C_trough_, 2 in uncomplicated [29, 31] and 1 in complicated [33] malaria (Additional file 1: Table S4). The required total C_trough_ values for uncomplicated [60] and complicated malaria [61] in pregnant women based on the current information in adults were 13–16 µg/ml, and 12.9–18.8 µg/ml, respectively. The therapeutic ranges of quinine for uncomplicated and complicated malaria during pregnancy, based on a study in Thailand [42] and France [60], were 14.257–17.5 [42] and 15.825–25.40 µg/ml [60], respectively. In addition to the curative treatment, the required total C_trough_ values for uncomplicated and complicated malaria during pregnancy (based on information in children [11] and adults [62]) were 2.6–4.37 and 7–14 µg/ml, respectively. However, total reported C_trough_ values in uncomplicated and complicated malaria were 2–3.9 [29, 31] and 7.1 [33] µg/ml, respectively (Additional file 1: Table S4). Therefore, none of the reported regimens provided adequate total quinine concentrations. Quinine dose adjustment may be required for pregnant women with uncomplicated and complicated malaria (Table 1). It is noted that the contribution of pharmacokinetics on quinine exposure remains inconclusive due to the limitation of sample size (See in Dose-dependent pharmacokinetics (Pregnant women)).

#### Elderly

With a single oral dose of 600 mg, plasma quinine concentration in elderly subjects was 10% lower than healthy adults [34] (Additional file 1: Table S3). There has been no pharmacokinetic study of this dose regimen in elderly with uncomplicated malaria. The C_trough_ in elderly, estimated from non-elderly adult patients with uncomplicated malaria (4.5 µg/ml) [59], is 4 µg/ml. Quinine plasma protein binding was unaffected by age [63]. An unbound C_trough_ concentration of 0.4 µg/ml in elderly is, thus, considered sufficient for uncomplicated malaria therapy. Plasma quinine concentration at steady-state in adult patients with severe malaria following standard regimen was 14 (10–20) µg/ml [64], which corresponds to 12.46 µg/ml in elderly. This C_trough_ is within the therapeutic range (10–20 µg/ml), and quinine dose adjustment may not be necessary for elderly (Table 1). Clinical study is required to support this argument.

The effects of anatomical and physiological differences in children and elderly, but not pregnant women, do not appear to influence the pharmacokinetics and clinical efficacy of quinine. Standard dose regimens of quinine are likely to be sufficient for effective malaria treatment in children (uncomplicated malaria) and elderly (uncomplicated and complicated malaria). Due to the expansion of plasma volume which may result in inadequate plasma quinine exposure, dose adjustment is likely to be required in pregnant women.

### Quinine adverse reactions and toxicity

#### Children

Eight out of 19 articles reported adverse reactions after quinine dosing, 3 in uncomplicated [11–13] and 5 in complicated [18–20, 24, 25] malaria. In uncomplicated malaria, no adverse reactions were reported following quinine doses ranging from 8.3 mg base (iv/im) to 11.85 mg base (ir), which produced C_max_ ranging from 4.43 to 9.86 µg/ml (0.31–0.69 µg/ml unbound C_max_) [11–13]. A C_max_ of over 20 µg/ml (1.4 µg/ml free C_max_) was reported in adult patients with cerebral malaria without toxicity [42]. The estimated toxic concentration in children based on healthy adult information is 28 µg/ml. The comparatively high toxic concentration reported in children was likely due to the increase in the fraction of free plasma drug in children.

Hypoglycaemia is a commonly reported adverse reaction to quinine in complicated malaria treatment (1–15%) [18, 19, 24, 25]. One article reported no association with hypoglycaemia in children with uncomplicated malaria [19]. The other 3 reported an association, but without supportive evidence on insulin levels [18, 24, 25]. Only 1 article reported quinine C_max_ of 25.9 µg/ml at the time of hypoglycaemia [18]. Two articles suggested that the standard glucose dose (3 mg/kg/min or 5% dextrose iv infusion) might be insufficient to correct hypoglycaemia in children [24, 25], instead suggesting a dose of 6 mg/kg/min [24]. The risk of quinine-induce hypoglycaemia is increased 3.2- (1.0–9.8) fold and should be a concern in children with pre-existing hypoglycaemia [24]. Since children with complicated malaria are likely to develop hypoglycaemia upon starting treatment, those that do present should have their blood glucose levels monitored throughout the treatment course.

QRS prolongation is one of the most serious concerns of quinine toxicity. One study reported a 10% incidence of QRS prolongation in children with complicated malaria receiving quinine treatment [20]; in this study, two patients experienced QRS prolongation leading to death, the quinine C_max_ of one of them was 16.9 µg/ml [20]. However, no correlation between QRS prolongation and free quinine concentration was found [20]. Although there was no correlation between QRS and free quinine concentration, plasma quinine concentration should be monitored.

Local irritation or pain at the injection site was commonly reported (12%) following im quinine administration but these symptoms resolved within 4 weeks [24]. The incidence of transient neurologic sequelae was 5% (1/18) with the standard quinine regimen in severe malaria [19]; however, there was no information on plasma quinine concentrations. Quinine C_max_ in fatal (15.0 ± 7.8 µg/ml, n = 2) and nonfatal (15.0 ± 3.9 µg/ml, n = 19) cases were similar, although the C_max_ in one fatal case was markedly high (25.9 µg/ml) [18]. About 5% (4/75) of children with complicated malaria receiving quinine had plasma concentrations over 25 µg/ml without toxicity [25]. The safety level of plasma quinine concentration (MTC) in complicated malaria could be as high as 25 µg/ml due to the higher level of plasma protein binding of quinine [20].

#### Pregnant women

Three out of 7 articles reported adverse reactions to quinine, 1 in uncomplicated [30] and 2 in complicated [32, 33] malaria. Mild-to-moderate tinnitus, headache, and epigastric pain occurred in uncomplicated malaria patients but they had recovered within 2–7 days [30].

Hypoglycaemia was the most commonly reported (50–100%) adverse reaction to quinine in complicated malaria [32, 33]. The relationship between plasma quinine concentrations and arterial blood pressure has been reported by Phillips et al. [33]. Quinine appears not to augment uterine contractions nor induce fetal distress.

#### Elderly

Adverse reactions to quinine were reported in two subjects after a single oral dose of 600 mg, one in an elderly patient with dizziness (4 µg/ml) and another in a young subject with tinnitus (4.3 µg/ml) [34]. Quinine-induced dizziness is of critical concern since it can lead to the fatal injury in the elderly [65]. Therefore, quinine dose administration in elderly, particularly by parenteral route, should be done carefully. Tinnitus was reversible without additional treatment. Since hypoglycaemia was the most commonly reported adverse reaction to quinine in other vulnerable groups, physicians should carefully prescribe quinine and advise patients of its side-effects.

### Pharmacokinetic analysis approaches

The average half-life based on NCA, CA, and pop-PK approaches in uncomplicated malaria were 10 [10], 10 [9], and 9.1 [14] h, respectively. The corresponding values in complicated malaria were 11.6 ± 3 [17–19], 12.0 ± 3.8 [20, 21, 23], and 16 ± 5.5 [24, 25] h. The volume of distribution vs. clearance reported using NCA and pop-PK in uncomplicated malaria were 1.6 l/kg vs. 1.48 l/h [10] and 1.12 l/kg vs. 1.12 l/h [14], respectively. The parameters reported in complicated malaria applying NCA, CA, and pop-PK were 1.36 ± 0.2 l/kg vs. 0.07 ± 0.01 l/h [17, 19], 0.80 ± 0.35 l/kg vs. 0.045 ± 0.015 l/h [20, 23], and 1.31 ± 0.10 l/kg vs. 0.063 ± 0.013 l/h [24, 25], respectively. The half-life, volume of distribution, and clearance reported based on the three approaches in uncomplicated malaria were comparable. However, the reported half-life and volume of distribution in complicated malaria based on CA were both slightly lower than those of NCA.

## Limitations and suggestions

The MIC or MCOC of quinine for *P. falciparum* malaria has not been reported since 2007 [55]. As the susceptibility of *P. falciparum* to quinine changes over time, inaccurate estimation of MIC and MCOC values may lead to inappropriate dose optimization in populations. The sample sizes used in most studies are small, which may not provide adequate power to detect small differences in the parameters under investigation, and thus, lead to incorrect data interpretation and conclusions. The study designs applied in most studies are not double-blind, or randomized controlled trials (RCT), which could result in bias influencing data interpretation and conclusions. Only a few studies applied statistical analysis to draw conclusions on the significant difference between the observed parameters. The heterogeneity of blood sampling frequency for pharmacokinetic investigations makes comparison among various groups or populations difficult. A conclusive understanding of the effect of a single factor (malaria disease, pregnancy status, age) on quinine pharmacokinetics is obscured by the intertwined nature of these factors in the studied populations. Further, pregnancy status (trimester periods) and age groups are not well defined in some studies. As quinine pharmacokinetics varies as a result of the physiological differences between children, elderly, and pregnant women, data obtained from such ill-defined subpopulations could lead to misguided conclusions. Conducting large clinical trials with a sufficient number of participants may have several limitations and ethical concerns in these vulnerable groups of malaria patients. In recent years, PBPK has emerged as a promising pharmacokinetic analysis tool which could be applied for accurate dose optimization for various drug classes with no requirement of large study sample sizes. PBPK is a mathematical model describing drug disposition in the human body based on prior knowledge from both in vitro and in vivo studies [66]. This model consists of various compartments corresponding to real anatomy and physiology of humans and can accurately predict optimal drug dosage regimens in various populations using a large number of virtual populations and applying prior knowledge from past clinical studies [66] (Additional file 1)

## Conclusion

The information of quinine pharmacokinetics in children, pregnant women, and the elderly with uncomplicated and complicated malaria are limited. Malaria infection and severity, routes of quinine administration, and nutritional status are the key factors that influence quinine systemic exposure and pharmacokinetics. The recommended dosages for both uncomplicated and complicated malaria are, in general, adequate for the elderly and children with uncomplicated malaria. In pregnant women with either uncomplicated or complicated malaria, and children with complicated malaria however, dose adjustment may be required. The discrepancies of the reported pharmacokinetics, particularly the volume of distribution and clearance, limit accurate dose optimization. Large clinical trials applying pop-PK or PBPK analysis would provide insight on the clinically relevant relationship between pharmacokinetics and clinical outcome parameters following various quinine dose regimens in these vulnerable populations.

## Key points

Standard dose regimens of quinine for the treatment of uncomplicated and complicated malaria optimized for the general population have been used in special population (i.e., children, pregnant women, and the elderly) without adjustment, even though these special populations have differences in their physiology and anatomy. Current standard dose regimens for malaria treatment for both uncomplicated and complicated malaria are sufficient for the elderly, but not for pregnant women. In addition to children, only the standard dosage regimen of quinine for uncomplicated malaria treatment is sufficient for malaria treatment. However, no standard dosage regimen of quinine for complicated malaria has been applied in children.

## Supplementary Information

**Additional file 1: Table S1**. Summary of quinine pharmacokinetic studies in children with malaria (uncomplicated malaria and complicated malaria). Pharmacokinetic parameters (Cmax and systemic exposure) are presented as mean + SD or mean or median (range) or median values. **Table S2**. Summary of quinine pharmacokinetic studies in pregnant women with malaria (uncomplicated malaria and complicated malaria). Pharmacokinetic parameters (Cmax and systemic exposure) are presented as mean + SD or mean or median (range) or median values. **Table S3**. Summary of quinine pharmacokinetic studies in the elderly. Pharmacokinetic parameters (Cmax and systemic exposure) are presented as mean+SD or mean or median (range) or median values. **Table S4**. In vitro quinine susceptibility (IC50) for Plasmodium falciparum isolates collected from Cambodia (2001-2007) and Thailand border (1998-2003).

## Data Availability

Not applicable.
