# Supplementary material for: Clinical pharmacokinetics of quinine and its relationship with treatment outcomes in children, pregnant women, and elderly patients, with uncomplicated and complicated malaria: a systematic review
Source: Malar J. 2022 Feb 10;21:41. doi: 10.1186/s12936-022-04065-1 (PMC8832728; doi:10.1186/s12936-022-04065-1)
Supplement: Supplementary file 1 — Additional file 1: Table S1. Summary of quinine pharmacokinetic studies in children with malaria (uncomplicated malaria and complicated malaria). Pharmacokinetic parameters (Cmax and systemic exposure) are presented as mean + SD or mean or median (range) or median values. Table S2. Summary of quinine pharmacokinetic studies in pregnant women with malaria (uncomplicated malaria and complicated malaria). Pharmacokinetic parameters (Cmax and systemic exposure) are presented as mean + SD or mean or median (range) or median values. Table S3. Summary of quinine pharmacokinetic studies in the elderly. Pharmacokinetic parameters (Cmax and systemic exposure) are presented as mean+SD or mean or median (range) or median values. Table S4. In vitro quinine susceptibility (IC50) for Plasmodium falciparum isolates collected from Cambodia (2001-2007) and Thailand border (1998-2003). [file 12936_2022_4065_MOESM1_ESM.docx]

**SUPPLEMENTARY**

Table S1 Summary of quinine pharmacokinetic studies in children with malaria (uncomplicated malaria and complicated malaria). Pharmacokinetic parameters (C_max_ and systemic exposure) are presented as mean+SD or mean or median (range) or median values.

| Reference | Study location/  Year | Study details | Regimen | PK analysis/Parameters | Main findings | |
| --- | --- | --- | --- | --- | --- | --- |
| Uncomplicated Falciparum Malaria | | | | | |  |
| Barennes H et al [1]. | Nigeria/1993 | -Intrarectal (n=7)  -Intramuscular (n=7)  -Intravenous (n=7) | - 12.8 mg salt (8 mg base)/kg ir q8h for 3 d  - 8 mg salt (4.74 mg base)/kg im q8h for 3 d  - 8 mg salt (4.74 mg base)/kg iv q8h for 3 d  (quinine gluconate) | Intrarectal  C_max_ 3 (2-4.8)* mg/l[Unbound C_max_, _plasma_: 0.3 (0.2-0.48)mg/l], C_48h_ 1.5* mg/l[Unbound C_48h,plasma_: 0.15 mg/l]  Intramuscular  C_max_ 3.2 (2.1-4.2)* mg/l[Unbound C_max_, _plasma_: 0.32 (0.21-0.42) mg/l], C_48h_ 2* mg/l[Free C_48h,plasma_: 0.20 mg/l]  Intravenous  C_max_ 5.1 (3.5-7.0)* mg/l[Unbound C_max_, _plasma_: 0.51 (0.35-0.70) mg/l], C_48h_ 3.57* mg/l[Free C_48h,plasma_: 0.36 mg/l] | 100% curative rate  (the reported MIC in Africans = 3 mg/lin whole-blood)  No 28d recrudescence recorded | |
| Barennes H et al [2]. | Nigeria/1996 | - Intravenous (n=5, 7.3 ±1.6 yr)  - Intramuscular (n=5, 10.6 ±1.3 yr,)  -Intrarectal (n=5, 6.2 ±1.9 yr) | -iv (5) 8 mg salt (4.74 mg base) /kg q8h for 3 d  -im (5) 8 mg salt (4.74 mg base) /kg q8h for 3 d  -ir (5) 20 mg salt (11.85 mg base) /kg q8h for 3 d  (quinine gluconate) | Intravenous  C_max_ 4.9 (0.5)* mg/l[Unbound C_max_, _plasma_: 0.49 (0.05) mg/l] , C_8h_ 3.1 (0.4)* mg/l[Free C_8h,plasma_: 0.31 (0.04) mg/l]  Intramuscular  C_max_ 3.3 (0.4)* mg/l[Unbound C_max_, _plasma_: 0.33 (0.04) mg/l], C_8h_ 2.1 (0.4)* mg/l[Free C_8h,plasma_: 0.21 (0.04) mg/l]  Intrarectal  C_max_ 4.6 (0.8)* mg/l[Unbound C_max_, _plasma_: 0.46 (0.08) mg/l], C_8h_ 2.8 (0.4)* mg/l[Free C_8h,plasma_: 0.28 (0.04) mg/l] | -100% curative rate  No 28d recrudescence recorded | |
| Treluyer JM et al.[3] | Gabon/1996 | -Malaria with full-nourished (n=7, 34±12) (21-58) months  -Malaria with malnourished (n=8, 21±16) (9-60) months | Loading dose16 mg base/kg (im), followed by 8 mg base/kg q12h  (quinine resorcine/quinine hydrochloride) | Full-nourished  QN: C_max_ 10.7 (2.9) mg/l[Unbound C_max_, _plasma_: 0.75 (0.2) mg/l], C_12h_ 5.3 (1.6) mg/l[Free C_12h,plasma_: 0.37 (0.11) mg/l]  Malnourished  QN: C_max_ 9.9 (3.1) mg/l[Unbound C_max_, _plasma_: 0.69 (0.22) mg/l], C_12h_ 3.3 mg/l[Unbound C_12h,plasma_: 0.23 mg/l] | Only one with positive *P. falciparum*. | |
| Barennes H et al [4]. | Nigeria/1996 | Group I (n=4, 6.5+1.3 (5-8) yr)  Group II (n=5, 9.4+5 (4-15) yr)  Group III (n=4, 7.6+4.6 (3-14) yr) | Group I: 8 mg salt (4.74 mg base)/kg (ir) q8h for 3 d  Group II: 13 mg salt (7.7 mg base)/kg (ir) q8h for 3 d  Groups III: 20 mg salt (11.85 mg base)/kg (ir) q12h for 3 d  (quinine gluconate) | Group I: C_max_: 2.6 (±1.8)* mg/l[Unbound C_max_, _plasma_: 0.26 (0.18) mg/l], C_8h_ 1.77 (0.27)* mg/l[Unbound C_8h,plasma_: 0.18 (0.03) mg/l]  Group-II: C_max_ 3.2 (1.5)* mg/l[Unbound C_max_, _plasma_: 0.32 (0.15) mg/l], C_8h_ 2.3 (0.03)* mg/l[Free C_8h,plasma_: 0.23 (0.03) mg/l]  Group III: C_max_ 5.1 (1.7)* mg/l[Free C_max_, _plasma_: 0.51 (0.17) mg/l], C_12h_ 1 (0.05)* mg/l[Free C_12h,plasma_: 0.10 (0.005) mg/l] | 100% curative rate  MIC in Africa = 2-3 mg/lwhole blood (3-5 mg/lplasma)  No 28d recrudescence recorded | |
| Barennes H et al [5]. | Burkina Faso/2002) | Group I (n=12, 91±28 months)  Group II (n=12, 96±37 months)  Group III (n=12,105±36 months)  Group IV (n=12, 92±36 months) | Group I: 8 mg base/kg QN (iv) over 4 h q8h for 2 d, followed by oral doses for 3 d  Group II: 8 mg base/kg QN (ir) q8h for 2 d, followed by oral doses for 3 d  Group-III: 8 mg base/kg Cinchona alkaloid mixture (iv) over 4 h q8h for 2 d, followed by oral doses for 3 d  Group-IV: 8 mg base/kg Cinchona alkaloid mixture (ir) over 4 h q8h for 2 d, followed by oral doses for 3 d  (quinine gluconate) | Group I:  QN: C_max_ 6.9 (1.9)* mg/l[Free C_max_, _plasma_: 0.69 (0.19) mg/l], C_8h_ 4.5 (1.7)* mg/l[Free C_8h,plasma_:0.45 (0.17) mg/l]  Group II  QN: C_max_ 3.5 (1.4)* mg/l[Free C_max_, _plasma_: 0.35 (0.14) mg/l], C_8h_ 3 (1.4)* mg/l[Free C_8h,plasma_: 0.30 (0.14) mg/l]  Group III  QN: C_max_: 5.2 (1.3)* mg/l[Free C_max_, _plasma_: 0.52 (0.13) mg/l], C_8h_ 4 (0.9)* mg/l[Free C_8h,plasma_: 0.40 (0.09) mg/l]  Group IV  QN: C_max_ 3.1 (1.6)* mg/l[Free C_max_, _plasma_: 0.31 (0.16) mg/l], C_8h_ 2.6 (1.3)* mg/l[Free C_8h,plasma_: 0.26 (0.13) mg/l] | 100% curative rate  No 28d recrudescence recorded | |
| Pussard E et al [6]. | Burkina Faso/2004 | Group I n=10, 99 (24-162) months, M:F: 1:1  Group II n=10, 72 (4-144) months, M:F: 7:3  Group III n=10, 60 (40-122) months, M:F: 2:3  Group IV n=10, 60 (24-120) months, M:F: 3:2  Group V n=10, 90 (36-146) months, M:F: 7:3  Group VI n= 10, 72 (30-120) months, M:F: 2:3 | Group-I: 12 mg salt (7.06 mg base) /kg (im) q 12h for 4 doses, followed po doses for 3 d  Group-II: 8 mg salt (4.74 mg base) (iv) over 4h q 8h, followed by po doses for 3 days;  Group-III: 8 mg salt (4.74 mg base) (ir) q 8h for 2 d, followed by po doses for 3 d  Group-IV: 16 mg salt (9.48 mg base)/kg (ir) q 8h for 2 d, followed by po doses for 3 d  Group-V: 12 mg salt (7.06 mg base) (ir) q 12h for 2 d, followed by po doses for 3 d  Group-VI: 20 mg salt (11.85 mg base) (ir) q 12h for 2 d, followed by po doses for 3 d  (quinine bichlorohydrate solution/quinine resorcine hydrochloride) | Group I  C_max_ 6.10 (4.5-10.4)* mg/l[Free C_max_, _plasma_: 0.61 (0.45-1.04) mg/l], C_8or12h_ 4.5 (3.1-6.7)* mg/l[Free C_8 or 12h,plasma_: 0.45 (0.31-0.67) mg/l], C_24h_ 5.7 (2.1-8.1)* mg/l[Free C_24h,plasma_: 0.57 (0.21-0.81) mg/l]  Group II  C_max_ 5.9 (3.9-6.7)*mg/l[Free C_max_, _plasma_: 0.59 (0.39-0.67) mg/l], C_8or12h_ 4.4 (2.9-5.4)* mg/l[Free C_8 or 12h,plasma_: 0.44 (0.29-0.54) mg/l], C_24h_ 6.35 (3.9-8.4)* mg/l [Free C_24h,plasma_: 0.64 (0.39-0.84) mg/l]  Group III  C_max_ 4.6 (2.1-6.2)* mg/l[Free C_max_, _plasma_: 0.46 (0.21-0.62) mg/l], C_8or12h_ 2.8 (0.9-5.3)* mg/l[Free C_8 or 12h,plasma_: 0.28 (0.09-0.53) mg/l], C_24h_ 3.7 (1.9-5.4)* mg/l [Free C_24h,plasma_: 0.37 (0.19-0.54) mg/l]  Group VI  C_max_ 5.1 (3.3-6.7)* mg/l[Free C_max_, _plasma_: 0.51 (0.33-0.67) mg/l], C_8or12h_ 2.7 (1.2-5.0)* mg/l[Free C_8 or 12h,plasma_: 0.27 (0.12-0.50) mg/l], C_24h_ 3.2 (1.3-6.6)* mg/l [Free C_24h,plasma_: 0.32 (0.13-0.66) mg/l]  Group V  C_max_ 5.1 (3.6-6.4)* mg/l[Free C_max_, _plasma_: 0.51 (0.36-0.64) mg/l], C_8or12h_ 4.2 (2.6-5.2)* mg/l[Free C_8 or 12h,plasma_: 0.42 (0.26-0.52) mg/l], C_24h_ 4.5 (2.7-6.8)* mg/l [Free C_24h,plasma_: 0.45 (0.27-0.68) mg/l]  Group VI  C_max_ 5.6 (4.2-8.5)* mg/l[Free C_max_, _plasma_: 0.56 (0.42-0.85) mg/l] , C_8or12h_ 4.2 (2.6-5.2)* mg/l[Free C_8 or 12h,plasma_: 0.42 (0.26-0.52) mg/l], C_24h_ 5 (2.5-8.5)* mg/l [Free C_24h,plasma_: 0.50 (0.25-0.85) mg/l] | 100% curative rate  No 28d recrudescence recorded | |
| Jouan MLE et al[7]. | Cameroon/2005 | n= 30, M:F: 17:13, 2.8+.7 (0.55-6.7) yr | 8.3 mg base/kg (po) q8h for 5 d (15 doses)  (quinine formiate) | C_50_ 6.6 (4.3-9.2) mg/l[Free C_50, plasma_: 0.46 (0.30-0.64) mg/l] | 100% curative rate  No 28d recrudescence recorded | |
| Kayumba PC et al [8]. | Rwanda/2008) | n=56, 6-59 months | 10 to 12 mg/kg (po) q8h for 7 d  (quinine pamoate) | C_max_,_ss_ 14.9 (10) mg/l[Free C_max_,_ss_: 1.04 (0.70) mg/l], C_8h, ss_ 10.4 (2.1) [8.6-13.3] mg/l[Free C_8h, ss_: 0.73 (0.15) (0.60-0.93) mg/l] | -100% curative rate  No recrudescence within 14d | |
| Kayitare E et al [9]. | Rawanda/2010 | n=56, M:F: 27:29, 28.5±16.1 (6-59) months | 8 mg base/kg (po) q 8h for 7 d  (quinine sulphate) | C_max_ 15.8 mg/l[Free C_max_: 1.11 mg/l], C_av,ss_ 10.4 (3.4) mg/l[Free C_av, ss_: 0.73 (0.24) mg/l], C_8h, ss_ 5.7 mg/l[Free C_8h, ss_: 0.40 mg/l] | -100% curative rate  -No recrudescence within 14d | |
| Complicated Falciparum Malaria | | | | | | |
| Frank S et al[10] | Papua New Guinea (Madang)/1982 | Group I n=7,33 (1-96) months  Group II n=8, 30 (5-48) months  Group III n=6, 41 (18-72) months | Group I: 14.6±1.8 mg salt (13.9±1.7 mg base)/kg (iv) over 4 h q12h for 5 d  Group II: 14.6±1.8 mg salt (13.9±1.7 mg base)/kg (im) q12h for 5 d  Group III: 14.6±1.8 mg salt (13.9±1.7 mg base)/kg (nasogastric tube) q12h for 5 d  (quinine dihydrochloride) | Group I  C_max_ 9.7 (3.7)** mg/l[Free C_max_, _plasma_: 0.69 (0.26) mg/l], C_24h_ (n=3) 3.1 (0.9)** mg/l[Free C_24h,plasma_: 0.23 (0.06) mg/l]  Group II  C_max_ 10.6 (4.4)** mg/l[Free C_max_, _plasma_: 0.76 (0.31) mg/l], C_12h_ 4.3 (2.5)** mg/l[Free C_24h,plasma_: 0.31 (0.18) mg/l]  Group III  C_max_ 10.3 (5.7)** mg/l[Free C_max_, _plasma_: 0.77 (0.41) mg/l], C_18h_  5.2 (4.8)** mg/l[Free C_24h,plasma_: 0.37 (0.34) mg/l] | **Suggested serum-quinine= 10 mg/l  **Reported MIC in Thailand: 20 mg/l(1983).  No reported efficacy. | |
| Waller D et al [11]. | Gambia/1988 | n=21, 4.5+2.1 (1.5-8) yr | Loading dose 20 mg salt (16.7 mg base)/kg (im), followed by 10 mg salt (8.3 mg base) (im) q12h (when patients can eat then 10 mg salt (8.3 mg base) po q12h to complete 5 d  (quinine dihydrochloride) | C_max_ 15.4 (4.5) (4.0-25.9) mg/l[Free C_max_, _plasma_: 0.85 (0.25) (0.22-1.42) mg/l], C_trough_ 11.1-13.1 mg/l[Free C_trough_: 0.61-0.72 mg/l]  *Fatal cases (n=2):* C_max_ 15.0 (7.8) mg/l[Free C_max_, _plasma_: 0.83 (0.43) mg/l], C_max_, death (n=1) 25.9 mg/l[Free C_max_, _plasma_: 1.42 mg/l]  *Survival cases (n=19):* C_max_ 15.0 (3.9) mg/l[Free C_max_, _plasma_: 0.83 (0.21) mg/l] | Survival rate: 90.48% (19/21)  -2 died:  1 with hypoglycemia (QN> 25.9 mg/l), no abnormality of pulse rate and blood pressure)  1 with QN <10 mg/lduring the first dose. | |
| Pasvol G et al [12]. | Kenya/1989-1990 | High-dose (iv) n=18, 4.3+3.2 yr  Low-dose (iv) n= 21, 2.8+1.6 yr  im n=20, 3.4+2 yr | High-dose (iv): Loading dose 20 mg salt (16.35 mg base)/kg (iv) over 2h, followed by 10 mg salt (8.17 mg base) /kg (iv) over 2 h q12h  Low-dose (iv): Loading dose 10 mg salt (8.3 mg base) /kg (iv) over 4h, followed by 5 mg salt ( 4 mg base) /kg (iv) over 4 h q12h  im: Loading dose 20 mg salt (16.7 mg base)/kg (im), followed by 10 mg salt (8.3 mg base) (im) q12h  *When children can eat 12 or 6 mg salt/kg po q12h to complete 5 d | High-dose (iv) (n=15)  C_max_ 15.3 (5.5) mg/l[Free C_max_: 0.84 (0.31) mg/l], C_48h_ 6.9 mg/l[Free C_48h_: 0.39 mg/l]  Low-dose (iv) (n=13)  C_max_ 9.70 (5.3) mg/l[Free C_max_: 0.53 (0.29) mg/l] ], C_48h_ 3.7 mg/l[Free C_48h, plasma_: 0.20 mg/l]  Im (n=15)  C_max_ 15.3 (7.6) mg/l[Free C_max_: 0.84 (0.42) mg/l] ], C_48h_ 7.7 mg/l[Free C_48h_: 0.42 mg/l] | Survival rate: 86% (51/59).  . | |
| Henbroek MBV et al [13]. | Gambia/1991 | Group I n=8, < 24 months  Group II n=12, > 24 months | Group I: Loading dose 20 mg salt (16.35 mg base)/kg (iv) over 2h, followed by 10 mg salt (8.17 mg base) /kg (iv) over 2 h q12h  Group II: Loading dose 20 mg salt (16.7 mg base)/kg (im), followed by 10 mg salt (8.3 mg base) (im) q12h  *When patients can eat then 10 mg salt (8.3 mg base) po q 12 to complete 5 d  (quinine dihydrochloride) | Group I (n=6)  C_max_ 16.3 (3.6) (8.8-22.5) mg/l[Free C_max_, _plasma_: 0.90 (0.20) (0.48-1.24) mg/l]  Group II (n=10)  C_max_ 17.4 (2.4) (14.7-20.8) mg/l[Free C_max_, _plasma_: 0.96 (0.13) (0.81-1.14) mg/l] | -Survival rate: 75.86% (22/29).  -Curative rate: 100%  -2/20 (10%) QRS prolongation at 2 h (QN 12.5 & 12.4 mg/l)  -1/2 (50 %) died at 4 h (QRS increased 30% of baseline, 16.9 mg/l), without correlation between free QN and QRS at 4 h | |
| Barennes H et al [14]. | Nigeria/1992 | Group I n=55, 56.4±3.5 months  Group II n=11, 98.6 ±1.1 months | Group I: 20 mg salt (11.9 mg base)/kg (ir) q 12h for 3 d  Group II: 12.5 mg salt (7.41 mg base)/kg (im) q 12h for 3 d  (quinine gluconate) | Group I (n=15)  C_max_ 4.9 (0.6) mg/l[Free C_max_, _plasma_: 0.25 (0.03) mg/l], C_8h_ 4 mg/l[Free C_8h_: 0.22 mg/l], C_24h_: 4 mg/l[Free C_24h_: 0.22 mg/l], C_36h_ 3.5 mg/l[Free C_36h_: 0.19 mg/l]  Group II (n=5)  C_max_ 9.1 (1.2) mg/l[Free C_max_, _plasma_: 0.50 (0.07) mg/l], C_8h_ 7.5 mg/l[Free C_8h_: 0.41 mg/l], C_24h_ 8 mg/l[Free C_24h_: 0.44 mg/l], C_36h_ 9.1 mg/l[Free C_36h_: 0.50 mg/l] | -Survival rate: 100%  -Curative rate: 100%.  -Parasite clearance time (h)= 46.5 ±5.7 (ir), 27.4±3.6 (im).  - Fever clearance time (h)= 48.6±2.7 (ir), and 35.9±2.2 (im).  - Relative bioavailability of ir/im route= 37-40% | |
| Newton CJC et al [15]. | Kenya/1993 | Group I n=15, 39.1+22.6 months  Group II n=14, 33.1+19.65 (8-84) months, M:F: 4:3 | Group I: loading dose 20 mg salt (16 mg base)/kg (im), followed by 10 mg salt (8 mg base) (im) q 12h  Group II: Loading dose 20 mg salt (16 mg base)/kg plus sulfadoxine(S)/pyrimethamine (P) (1.25 mg/kg for S and 25 mg/kg for P) (im), followed by 10 mg salt (8 mg base) (im) q 12h for 6 doses  (quinine dihydrochloride) | Group I  C_max_ 13.5 (±2.8)* mg/l[Free C_max_, _plasma_: 0.80 (0.17) mg/l]  Group II  C_max_ 15.9 (4.4)* mg/l[Free C_max_, _plasma_: 0.95 (0.26) mg/l] | -Survival rate (QN): 92.5%  -Survival rate (QN/SP): 95%  -Curative rate (QN): NR  -Curative rate (QN/SP): NR  -Fever clearance (QN): 25.1 (16)h  -Fever clearance (QN/SP): 20.1 (18.86)h  -Parasite clearance (QN): 49.5 (15.22)h  -Parasite clearance: (QN/SP): 45.9 (14.61)h | |
| Pussard E et al [16]. | Nigeria/1996 | Group I healthy, n=9, 44.8 + (20.9) months, M:F: 9:1)  Group-II (malaria): n=10 (age: 36.5 (10.1) months, M:F: 3:7)  Group-III (malnutrition): n=10 (age: 34.2 (7.8) months, M:F: 2:3)  Group-IV (malnutrition with malaria): n=10 (age: 41.8 (9.0) months, M:F: 2:3) | Group I & III: Single dose of 8 mg salt (4.7 mg base)/kg iv over 4h q8h  Group II & IV: 8 mg salt (4.7 mg base)/kg (iv) over 4h q 8h until po 10 mg salt/kg  (quinine gluoconate) | Group I (healthy)  C_max_ 3.0 (2.1) mg/l[Free C_max_, _plasma_: 0.30 (0.21) mg/l]  Group II (malaria)  C_max_ 6.6 (3.0) mg/l[Free C_max_, _plasma_: 0.36 (0.17) mg/l], C_12h_ 9.8 (2.3) mg/l[Free C_12h_: 0.54 (0.13) mg/l], C_16h_ 7.6 (4.0) mg/l[Free C_16h_: 0.42 (0.22) mg/l], C_24h_ 7.7 (2.6) mg/l[Free C_24h_: 0.42 (0.14) mg/l]  Group III (malnutrition)  C_max_ 8.5 (4.7) mg/l[Free C_max_, _plasma_: 0.85 (0.47) mg/l]  Group IV (malnutrition with malaria)  C_max_ 7.7 (2) mg/l[Free C_max_, _plasma_: 0.42 (0.11) mg/l], C_12h_ 9.6 (2.7) mg/l[Free C_12h_: 0.53 (0.15) mg/l], C_16h_ 6.8 (2.6) mg/l[Free C_16h_: 0.37 (0.14) mg/l], C_24h_ 7.8 (2.4) mg/l[Free C_24h_: 0.39 (0.13) mg/l] | -Survival rate: 100%  -Curative rate: 100%.  -Time to regain consciousness 35 ±9 h (group II), 39±11 h (group IV)  - Fever clearance time =36±8 h (group II), 44±15 h (group IV)  -Time to 50% parasite clearance =  29±21 h (group II),18±12 h (group IV) | |
| Krishna S et al [17]. | Ghana/1997-1999 | Malaria-associated lactic acidosis (n=120), 38.6±23.3 months, 92 (74%) history of antimalarial treatment (amodiaquine 1, artesunate 1, chloroquine 80, unspecified 10), 42 received phenobarbitone during admission | Loading dose 20 mg salt (16 mg base)/kg (im), followed by 10 mg salt (8 mg base) (im) q12h  (quinine dihydrochloride) | C_max_: NA, C_trough_: NA | -6/120 (5%) plasma QN >30 mg/lwithout toxicities | |
| Hendriksen ICE et al [18]. | Tanzania/2009-2010 | n=75, 2.4 (0.33-8.1) yr,  <2 yr=28 (37%)  > 2 yr =47 (63%)  -69/75 included in study, 41/75 history of antimalarials pretreatment (quinine 8, amodiaquine 5, artemether-lumefantrine 17, S/P 10, S/P + artemether-lumefantrine 1) | -Loading dose 20 mg salt (16 mg base) /kg (im), followed by 10 mg salt (8 mg base) (im) q12h (n=69 or 92%) for 2 doses, followed by a full course of oral artemether-lumefantrine  -10 mg salt (8 mg base) (im) q12h (n=6 or 8%) for 3 doses, followed by a full course of oral artemether-lumefantrine  (quinine dihydrochloride) | Observation:  C_max_ 13.4 (7.2-24.8) mg/l[Free C_max_, _plasma_: 0.74 (0.40-1.36) mg/l], C_predose_: 6.90 (0.976-14.9) mg/l[Free C_predose_: mg/l], C_postdose_: 0.85-33.8 mg/l[Free C_postdose_: mg/l]  Prediction:  Simulated C_max_ (with loading dose) 12.6 (5.6-28.4) mg/l  [Free C_max_, _plasma_: 0.69 (0.31-1.56) mg/l], simulated C_max_ (without loading dose) 6.32 (2.8-14.2) mg/l[Free C_max_, _plasma_: 0.32 (0.15-0.78) mg/l]. | -Survival rate: 82.67%.  -Curative rate: NR  -12/13 fatal cases (loading dose) similar QN exposure to survivor cases (1 did not receive loading dose)  -4/75 (5%) QN > 25 mg/l, without toxicity | |
| Kawashiokor without Malaria | | | | | | |
| Salako et al. [19]. | Nigeria/1989 | Group I (healthy) n=7, 2.2 +0.6 (1.5-3) yr, M:F:3:4  Group II (kawashiokor), n=6 2.1+0.4) (1.25-2.5) yr, M:F: 1:2 | Single dose of 10 mg salt (8.33 mg base) po  (quinine hydrochloride) | Group I  C_max_ 2.4 (0.3) mg/l[Free C_max_, _plasma_: 0.24 (0.03) mg/l], C_36h_ 0.15 mg/l[Free C_36h_: 0.015 mg/l]  Group II  C_max_ 1.7 (0.5) mg/l[Free C_max_, _plasma_: 0.17 (0.05) mg/l], C_36h_ 0.45 mg/l[Free C_36h_: 0.045 mg/l] | -No recorded efficacy. | |

AUC: area under the plasma drug concentration-time curve; C_max_: maximum plasma concentration; C_i_: trough plasma concentration at time i; CL:total clearance; CL/F: the total clearance uadjusted by bioavailability; F: Absolute bioavailability; NCA: non-compartmental analysis; T_max_: time to reach maximum plasma concentration; T_1/2_: elimination half-life; V_d_/F: apparent volume of distribution unadjusted by bioavailability

V_d_: apparent volume of distribution

Table 2 Summary of quinine pharmacokinetic studies in pregnant women with malaria (iuncomplicated malaria and complicated malaria). Pharmacokinetic parameters (C_max_ and systemic exposure) are presented as mean +SD or mean or median (range) or median values.

| Reference | Study location/Year | Study subjects (n) | Regimen | PK analysis/Parameters | Main findings |
| --- | --- | --- | --- | --- | --- |
| Uncomplicated Falciparum Malaria | | | | | |
| Abdelrahim II et al [20]. | Eastern Sudan (New Halfa)/2007 | -2^nd^/3^rd^ trimester pregnant women (n=16)  -Non-pregnant women (n= 7) | 10 mg salt/kg (iv over 2 h), followed by 1.6 mg (im) artemether at 12 & 18h and once-daily for 4 d  (quinine hydrochloride) | Pregnant women  QN: C_max_ 38 +33 µM [Free C_max_, _plasma_: 0.99 (±0.86) mg/l]  Non-pregnant women  QN: C_max_ 27+5.7 µM [Free C_max_, _plasma_: 0.95 (±0.20) mg/l] | No reported clinical efficacy |
| Mirghani RA et al [21]. | Eastern Sudan (New Halfa)/2010 | -Pregnant women (n=9)  -Non-pregnant women (n=8)  (cross-over design, with one-week washout period) | Phase A: 10 mg salt/kg (iv over 2 h), followed by 1.6 mg artemether (im) at 12 & 18 h and once-daily for 4 d  Phase B: 10 mg salt/kg (iv over 2 h)  (quinine hydrochloride) | Pregnancy during acute phase malaria  QN: C_max_ 30 (19-49) µM [Free C_max_, _plasma_: 0.78 (0.49-1.27) mg/l]  Pregnancy during convalescence  QN: C_max_ 20 (16-24) µM [Free C_max_, _plasma_: 0.84 (0.67-1.01) mg/l]  Non-pregnancy during acute phase malaria  QN: C_max_ 26 (21-33) µM [Free C_max_, _plasma_: 0.92 (0.74-1.17) mg/l]  Non-pregnancy during convalescence  QN: C_max_ 19.9 (15- 26.4) µM [Free C_max_, _plasma_: 0.96 (0.72-1.27) mg/l] | 100% curative rate |
| Kloprogge F et al [22]. | Uganda/2008 | -2^nd^ trimester pregnant women (N=12)  -3^rd^ trimester pregnant women (N=10) | 10 mg salt/kg (8.3 mg base) po q 8h for 7d  (quinine sulphate) | All  C_max_ 4 (2.4-7.9) mg/l[Free C_max_, _plasma_: 0.32 (0.24-0.63) mg/l], C_168h_ 3 mg/l[Free C_168h, plasma_: 0.24 mg/l], C_192h_: 2 mg/l[Free C_192h, plasma_: 0.16 mg/l]  2^nd^ trimester pregnancy  C_max_ 3.9 (2.5-6.6) mg/l[Free C_max_, _plasma_: 0.31 (0.2-0.53) mg/l]  3^rd^ trimester pregnancy  C_max_ 4.1 (2.4-7.9) mg/l[Free C_max_, _plasma_: 0.33 (0.19-0.63) mg/l] | No reported clinical efficacy |
| Kayentao K et al [23]. | Mali (Sikasso)/2010-2011 | 2^nd^ / 3^rd^ trimester pregnant women (n=7):  -QN + Nevirapine-based ART (n=6)  -QN alone (n=1) | 10 mg base/kg (total=600 mg base) q8h (po) for 7 d  (quinine sulphate) | QN + Nevirapine-based ART  QN: C_0-2d_ 5.3 (3.6-6.2) mg/l, C_0-2d, free_ 0.7 (0.6-0.9) mg/l, C_3-6 d_ 4.4 (3.6-6.2) mg/l, C_3-6d, free_ 0.70 (0.60-1.0) mg/l  QN alone  QN: C_0-2d_ 7.0 (6.5-7.5) mg/l, C_0-2d, free_ 1.7 (1.6-1.8) mg/l, C_3-6d_ 10.7 (10.0-11.0) mg/l, C_3-6d, free_: 2.0 (1.7-2.1) mg/l | -Mild-to-moderate tinnitus, headache, epigastric pain, recovered within 2-7 d.  -28d PCR-corrected cure rate 100% |
| Tarning J et al [24]. | Uganda/2013) | 2^nd^ Trimester (n=11) & 3^rd^ Trimester (n=10) | 10 mg salt/kg (8.3 mg base) q8h for 7 d (po)  (quinine sulphate) | C_max_ 4.5 (2.5-8) mg/l[Free C_max_, _plasma_: 0.36 (0.2-0.64) mg/l], C_d7_ 3.9 (1-7.7) mg/l[Free C_d7_: 0.31 (0.08-0.62) mg/l] | -42-day curative rate 97.6% |
| Complicated Falciparum Malaria | | | | | |
| Looareesuwan S et al. [25]. | Thailand (Thai-Cambodian border, Chanthaburi)/1982-1983 | 3^rd^ Trimester pregnant women (n=12) | Loading dose 20 mg salt/kg (16.7 mg base) (iv over 4 h), followed by 10 mg salt (8.3 mg base)/kg q8h  (quinine dihydrochloride) | C_max_ 10.4 (6.9-18.7) mg/l[Free C_max_, _plasma_: 0.52 (0.35-0.93) mg/l] | 91.67 % curative rate (11 out of 12) |
| Philip R.E. et al. [26]. | Thailand (Thai-Cambodian border, Chanthaburi)/1986/NR | -2^nd^ /3^rd^ Trimester pregnant women (n=10)  -At delivery (n=8)  -During breast feeding (n=30) | Loading dose 20 mg salt/kg (8.3 mg base) (iv over 4 h), followed by 10 mg salt (8.3 mg base)/kg q8h  (quinine dihydrochloride) | 2^nd^ /3^rd^ Trimester pregnant women  C_max_  10.4 (6.9-18.7) mg/l[Free C_max_, _plasma_: 0.52 (0.35-0.93) mg/l]  At delivery  C_cord_ 2.4 (1-4.6) mg/l[Free C_cord, plasma_: 0.6 (0.25-1.15) mg/l], Simulated C_avg, mother_: 7.1 mg/l[Free C_avg, mother_: 0.35 mg/l], C_foetal_ 2.8 mg/l[Free C_foetal, plasma_: 0.72 mg/l] | No reported clinical efficacy |

AUC: area under the plasma drug concentration-time curve; C_max_: maximum plasma concentration; C_i_: trough plasma concentration at time i; CL:total clearance; CL/F: the total clearance uadjusted by bioavailability; F: Absolute bioavailability; NCA: non-compartmental analysis; T_max_: time to reach maximum plasma concentration; T_1/2_: elimination half-life; V_d_/F: apparent volume of distribution unadjusted by bioavailability

V_d_: apparent volume of distribution

Table 3 Summary of quinine pharmacokinetic studies in the elderly. Pharmacokinetic parameters (C_max_ and systemic exposure) are presented as mean+SD or mean or median (range) or median values.

| Reference | Study location/Year | Study details | Regimen | PK analysis/Parameters | Main findings |
| --- | --- | --- | --- | --- | --- |
| Uncomplicated Falciparum Malaria | | | | | |
| Wanwimolruk S et al [27]. | New Zealand/1991 | -Healthy young subjects (n=12)  Age: 24 (30-35) yrs  -Healthy elderly subjects (n=8)  Age: 70 (65-78) yrs | 600 mg salt (497 base) (po)  -quinine sulphate | Healthy young subjects  C_max_ 5.6+1.2 mg/l[Free C_max_, _plasma_: 0.828 (±0.18) mg/l]  Healthy elderly subjects  C_max_ 5+1.3 mg/l[Free C_max_, _plasma_: NA] | -Tinnitus at 4.3 mg/l, dizziness at 4 mg/l  -Significant differences in t_1/2_, AUC_0-48h_, CL/F |
| Dyer JR et al [28]. | Australia/1994 | -Non-diabetic subjects (n=10)  Age: 63. ±6.5 yrs  -Type 2 diabetic subjects (n=12)  Age: 62.7±7.6 yrs | 600 mg salt (497 base) (po)  (quinine sulphate) | Non-diabetic subjects  C_max_ 3.4+0.8 mg/l[Free C_max_, _plasma_: NA]  Type 2 diabetic subjects  C_max_ 3.7+0.8 mg/l[Free C_max_, _plasma_: NA] | -No adverse events |

AUC: area under the plasma drug concentration-time curve; C_max_: maximum plasma concentration; C_i_: trough plasma concentration at time i; CL:total clearance; CL/F: the total clearance uadjusted by bioavailability; F: Absolute bioavailability; NCA: non-compartmental analysis; T_max_: time to reach maximum plasma concentration; T_1/2_: elimination half-life; V_d_/F: apparent volume of distribution unadjusted by bioavailability

V_d_: apparent volume of distribution

Table 4 *In vitro* quinine susceptibility (IC_50_) for *Plasmodium falciparum* isolates collected from Cambodia (2001-2007) and Thailand border (1998-2003)

| Thailand border (Thai-Cambodia and Thai-Myanmar) | | Western Cambodia | | Eastern Cambodia | |
| --- | --- | --- | --- | --- | --- |
| Year | GMIC_50_  mean ± 95%CI (nM) | Year | GMIC_50_ (nM)  (range) | Year | GMIC_50_ (nM)  (range) |
| 1998 | 178.1 (121.1-262.4) | 2001 | 164.6 (28.8-583.5) | 2001 | 83.6 (12-693.6) |
| 2000 | 243.4 (163.7-361.8) | 2002 | 93.6 (20.5-464.8) | 2002 | 60.6 (10.3-440.2) |
| 2002 | 184.7 (152.1-224.5) | 2003 | 103.2 (15.1-538) | 2003 | 69.8 (15.3-401.3) |
| 2003 | 136 (115.2-160.4) | 2004 | 226.6 (49.3-530.1) | 2004 | 79.8 (14.5-284.1) |
|  |  | 2005 | 230.3 (102.9-557.3) | 2005 | NR |
|  |  | 2006 | 163.8 (58.5-496.9) | 2006 | 112.8 (22.2-492.2) |
|  |  | 2007 | 301.8 (104.1-715.6) | 2007 | 249.1 (106.8-630.3) |

GMIC_50_: geometric mean of IC_50_; IC_50_: drug concentration corresponding to inhibit 50% of parasite.

References

[1] Barennes H, Pussard E, Mahaman Sani A, Clavier F, Kahiatani F, Granic G, et al. Efficacy and pharmacokinetics of a new intrarectal quinine formulation in children with Plasmodium falciparum malaria. Br J Clin Pharmacol. 1996;41:389-95.

[2] Barennes HV, F.; Clavier, F.; Pussard, E. Pharmacokinetics of quinimax(R) suppositories in children with malaria a preliminary study. Clin Drug Investig. 1999;17:5.

[3] Treluyer JM, Roux A, Mugnier C, Flouvat B, Lagardere B. Metabolism of quinine in children with global malnutrition. Pediatr Res. 1996;40:558-63.

[4] Barennes HP, A.; Sani AM.; Clavier, F.; Henzel, D.; Kahiatani, F.; Granic, G.; Lavinet, L.; Verdier, F. Intrarectal Quinimax® (a Combination of Cinchona Alkaloids) Administered at 3 Different Dosages to Children with Plasmodium falciparum Malaria in Niger. Clin Drug Investig. 1996;11:5.

[5] Barennes H, Sterlingot H, Nagot N, Meda H, Kabore M, Sanou M, et al. Intrarectal pharmacokinetics of two formulations of quinine in children with falciparum malaria. Eur J Clin Pharmacol. 2003;58:649-52.

[6] Pussard E, Straczek C, Kabore I, Bicaba A, Balima-Koussoube T, Bouree P, et al. Dose-dependent resorption of quinine after intrarectal administration to children with moderate Plasmodium falciparum malaria. Antimicrob Agents Chemother. 2004;48:4422-6.

[7] Le Jouan M, Jullien V, Tetanye E, Tran A, Rey E, Treluyer JM, et al. Quinine pharmacokinetics and pharmacodynamics in children with malaria caused by Plasmodium falciparum. Antimicrob Agents Chemother. 2005;49:3658-62.

[8] Kayumba PC, Twagirumukiza M, Huyghebaert N, Ntawukuliryayo JD, van Bortel L, Vervaet C, et al. Taste-masked quinine sulphate pellets: bio-availability in adults and steady-state plasma concentrations in children with uncomplicated Plasmodium falciparum malaria. Ann Trop Paediatr. 2008;28:103-9.

[9] Kayitare E, Vervaet C, Mehuys E, Kayumba PC, Ntawukulilyayo JD, Karema C, et al. Taste-masked quinine pamoate tablets for treatment of children with uncomplicated Plasmodium falciparum malaria. Int J Pharm. 2010;392:29-34.

[10] Shann F, Stace J, Edstein M. Pharmacokinetics of quinine in children. J Pediatr. 1985;106:506-10.

[11] Waller D, Krishna S, Craddock C, Brewster D, Jammeh A, Kwiatkowski D, et al. The pharmacokinetic properties of intramuscular quinine in Gambian children with severe falciparum malaria. Trans R Soc Trop Med Hyg. 1990;84:488-91.

[12] Pasvol G, Newton CR, Winstanley PA, Watkins WM, Peshu NM, Were JB, et al. Quinine treatment of severe falciparum malaria in African children: a randomized comparison of three regimens. Am J Trop Med Hyg. 1991;45:702-13.

[13] van Hensbroek MB, Kwiatkowski D, van den Berg B, Hoek FJ, van Boxtel CJ, Kager PA. Quinine pharmacokinetics in young children with severe malaria. Am J Trop Med Hyg. 1996;54:237-42.

[14] Barennes H, Kahiatani F, Pussard E, Clavier F, Meynard D, Njifountawouo S, et al. Intrarectal Quinimax (an association of Cinchona alkaloids) for the treatment of Plasmodium falciparum malaria in children in Niger: efficacy and pharmacokinetics. Trans R Soc Trop Med Hyg. 1995;89:418-21.

[15] Newton CR, Winstanley PA, Watkins WM, Mwangi IN, Waruiru CM, Mberu EK, et al. A single dose of intramuscular sulfadoxine-pyrimethamine as an adjunct to quinine in the treatment of severe malaria: pharmacokinetics and efficacy. Trans R Soc Trop Med Hyg. 1993;87:207-10.

[16] Pussard E, Barennes H, Daouda H, Clavier F, Sani AM, Osse M, et al. Quinine disposition in globally malnourished children with cerebral malaria. Clin Pharmacol Ther. 1999;65:500-10.

[17] Krishna S, Nagaraja NV, Planche T, Agbenyega T, Bedo-Addo G, Ansong D, et al. Population pharmacokinetics of intramuscular quinine in children with severe malaria. Antimicrob Agents Chemother. 2001;45:1803-9.

[18] Hendriksen IC, Maiga D, Lemnge MM, Mtove G, Gesase S, Reyburn H, et al. Population pharmacokinetic and pharmacodynamic properties of intramuscular quinine in Tanzanian children with severe Falciparum malaria. Antimicrob Agents Chemother. 2013;57:775-83.

[19] Salako LA, Sowunmi A, Akinbami FO. Pharmacokinetics of quinine in African children suffering from kwashiorkor. Br J Clin Pharmacol. 1989;28:197-201.

[20] Abdelrahim, II, Adam I, Elghazali G, Gustafsson LL, Elbashir MI, Mirghani RA. Pharmacokinetics of quinine and its metabolites in pregnant Sudanese women with uncomplicated Plasmodium falciparum malaria. J Clin Pharm Ther. 2007;32:15-9.

[21] Mirghani RA, Elagib I, Elghazali G, Hellgren U, Gustafsson LL. Effects of Plasmodium falciparum infection on the pharmacokinetics of quinine and its metabolites in pregnant and non-pregnant Sudanese women. Eur J Clin Pharmacol. 2010;66:1229-34.

[22] Kloprogge F, Jullien V, Piola P, Dhorda M, Muwanga S, Nosten F, et al. Population pharmacokinetics of quinine in pregnant women with uncomplicated Plasmodium falciparum malaria in Uganda. J Antimicrob Chemother. 2014;69:3033-40.

[23] Kayentao K, Guirou EA, Doumbo OK, Venkatesan M, Plowe CV, Parsons TL, et al. Preliminary study of quinine pharmacokinetics in pregnant women with malaria-HIV co-infection. Am J Trop Med Hyg. 2014;90:530-4.

[24] Tarning J, Kloprogge F, Dhorda M, Jullien V, Nosten F, White NJ, et al. Pharmacokinetic properties of artemether, dihydroartemisinin, lumefantrine, and quinine in pregnant women with uncomplicated plasmodium falciparum malaria in Uganda. Antimicrob Agents Chemother. 2013;57:5096-103.

[25] Looareesuwan S, Phillips RE, White NJ, Kietinun S, Karbwang J, Rackow C, et al. Quinine and severe falciparum malaria in late pregnancy. Lancet. 1985;2:4-8.

[26] Phillips RE, Looareesuwan S, White NJ, Silamut K, Kietinun S, Warrell DA. Quinine pharmacokinetics and toxicity in pregnant and lactating women with falciparum malaria. Br J Clin Pharmacol. 1986;21:677-83.

[27] Wanwimolruk S, Chalcroft S, Coville PF, Campbell AJ. Pharmacokinetics of quinine in young and elderly subjects. Trans R Soc Trop Med Hyg. 1991;85:714-7.

[28] Dyer JD, ME.; Giele, C.; Annus, T.; Garcia-webb, P.; Robson, J. The pharmacokinetics and pharmacodynamics of quinine in the diabetic and non-diabetic elderly. Br J Clin Pharmacol. 1994;38:8.
